# Towards a domain-general mechanistic model of fatigue: application to Functional Neurological Disorder

**DOI:** 10.1098/rstb.2024.0462

**Published:** 2026-09-17

**Authors:** Glenn R. Wylie

**Affiliations:** ^1^ Rocco Ortenzio Neuroimaging Center, Kessler Foundation West Orange Campus, West Orange, NJ, USA; ^2^ Physical Medicine and Rehabilitation, Rutgers Biomedical and Health Sciences, Newark, NJ, USA; ^3^ US Department of Veterans Affairs, East Orange, NJ, USA

**Keywords:** fatigue, functional neurological disorder, basal ganglia

## Abstract

Fatigue is a central symptom in individuals with functional neurological disorder (FND), yet few studies have investigated fatigue in this population. While this may be surprising, given the importance of fatigue in FND, it is nevertheless understandable, given that fatigue has historically been difficult to study. However, the tools of cognitive neuroscience have allowed researchers to begin to understand fatigue as never before. Here, I review recent advances in the field of fatigue research, ranging from experimental design to objective and subjective measures of fatigue to a network of brain areas that has shown sensitivity to fatigue. Taken together, these metrics support the idea that fatigue is a signal the brain generates when the reward received no longer merits the effort required. I conclude by arguing that the application of this domain-general mechanistic model of fatigue, using objective and subjective dependent measures, is likely to provide important insights into the fatigue that is so pervasive in FND.

This article is part of the discussion meeting issue ‘Digital healthcare for the management of functional neurological disorders’.

## 1. Introduction

Fatigue is a central complaint for individuals with functional neurological disorder (FND): it is the most commonly reported symptom [1,2], and is also significantly associated with every symptom of FND (the core symptoms, and the psychiatric and associated symptoms) [1]. Treating fatigue in FND should therefore be a high priority. However, treatments for fatigue remain elusive, regardless of aetiology. This is because the development of effective treatments for any condition requires at least two things: (i) that we know what we are treating (that is, it is well-defined), and (ii) that we know how to change it (that is, it can be manipulated). For fatigue, neither of these is true; fatigue is notoriously difficult to define and manipulate [3], and because our manipulations of fatigue are weak, we are unable to effectively treat it. Recently, a mechanistic understanding of fatigue has begun to be developed, providing hope for the development of effective interventions for fatigue in the future.

Recent advances in this field have been made possible by the tools of cognitive neuroscience, such as functional magnetic resonance imaging. Previously, researchers used other means to attempt to understand fatigue, such as describing the populations affected. For example, the observation that Parkinson's disease (PD) is associated with high levels of fatigue led to the proposal that the basal ganglia play a central role in fatigue [4]. More recently, it has been shown that Chronic Fatigue Syndrome is associated with hypometabolism in several metabolic pathways [5]. These inferences are important and relevant to mechanistic models of fatigue. However, they do not themselves provide a mechanistic account of how basal ganglia degradation or hypometabolism results in the observed increases in fatigue. Others, recognizing that fatigue is multi-dimensional, have fractionated fatigue into smaller, more constrained categories. One example of this is the fractionation of fatigue into dimensions such as cognitive fatigue, physical fatigue and social fatigue (e.g. [6]). These types of distinctions can be useful. For example, fractionating fatigue in this way allows clinicians to know that a given individual or group is more prone to one type of fatigue than another (e.g. physical versus cognitive fatigue). However, this knowledge is of questionable value if the definition of fatigue remains disputed. Moreover, even if this concern is set aside, it does not move us closer to a mechanistic understanding of fatigue.

## 2. Components of fatigue

In attempting to understand the mechanisms of fatigue, it is helpful to consider what I will call the components of fatigue in order to distinguish ‘components’ from ‘dimensions.’ Furthermore, I consider them to be components rather than simply associated psychological constructs because manipulating these components results in changes in fatigue. Fatigue dimensions, as noted above, are different domains of fatigue or different systems that fatigue may affect. For example, cognitive fatigue affects the cognitive system, whereas physical fatigue affects the musculoskeletal system. Whether physical fatigue relies on at least some of the same mechanisms as cognitive fatigue is currently an open question. For example, physical fatigue involves muscle fatigue, but it also involves ‘central’ fatigue that is assumed to rely on neural mechanisms in the brain and is thought to preserve homeostasis by ensuring that the muscles are not overused (e.g. [7,8]). While it is possible to imagine that ‘central’ physical fatigue is distinct from cognitive fatigue, it would be more parsimonious if they shared at least some of the same mechanisms—meaning that these fatigue dimensions may not be completely distinct.

The experience of fatigue, regardless of the fatigue dimension, is assessed through interoception, i.e. assessment of an internal state. Over 250 assessment tools have been developed to assess fatigue [9], all of which ask respondents to look into themselves and answer a single question: ‘Do I feel fatigued?’ This central question is an interoceptive question, just as questions about an individual's pain or level of seity are interoceptive questions. Fortunately for the field of fatigue research, previous research has shown that these types of interoception rely on a network of areas, including the anterior insular cortex (e.g. [10–12]). This finding from other research domains suggests that the insula (especially the anterior insula) should be involved when people experience fatigue, and this prediction has been borne out by several studies [13–17]. Thus, while interoception is not a component of fatigue, we should nevertheless expect activation in brain areas involved in interoception when fatigue is experienced.

The first component of fatigue is motivation, or a lack of motivation. When we are motivated, we either do not feel fatigue or are able to set it aside and continue on despite the fatigue. By contrast, when we are unmotivated (or demotivated) or when we are bored, fatigue is a common symptom. When faced with a task that is tedious or odious, a task that we must force ourselves to do, we frequently find that we must overcome a feeling of fatigue in order to get started (indeed, overcoming an abrupt onset of fatigue is what we mean when we ‘force ourselves to do it’). By contrast, when we engage in a hobby or pastime that we enjoy, we find that we can lose ourselves in its pursuit. Time passes unnoticed, and does so, in part, because we continue working without being burdened by fatigue (this is referred to as ‘flow’).

Experimentally, it has been shown that when participants are more motivated, they experience less fatigue. This has been done by contrasting a condition in which participants are able to earn a monetary reward on each trial (in addition to being compensated for participating in the experiment) with another condition in which no extra monetary reward is possible. The underlying idea is that when participants are able to earn additional money, they will be more motivated than when they cannot. This type of paradigm has shown that participants report less fatigue when they are able to earn additional money (and are therefore more motivated) than when they cannot [18,19]—a result that indicates that increasing rewards lessens fatigue itself, rather than merely modifying participants’ willingness to tolerate fatigue. These experiments help clarify what is meant by motivation in this context: it is the balance between the amount of effort participants must expend and the reward they expect in return for that effort expenditure [20–22]. Let us take these in turn.

One of the components of motivation, and therefore also of fatigue, is effort. The more effortful a task is, the more quickly it fatigues us. This seems straightforward enough, but the amount of effort a given task requires is not always easy to predict. For example, in a study investigating fatigue in individuals with traumatic brain injury (TBI), we asked participants to perform a relatively difficult working memory task—the 2-back condition of the n-back working memory paradigm [23,24]—and a trivial control task—the 0-back condition of the n-back task, which is essentially a letter-recognition task [25]. We included 31 participants who had sustained a TBI and 17 healthy control participants who were matched to the TBI group by age, education and gender. All participants performed four blocks of each task, rating their fatigue on a visual analogue scale of fatigue (VASF) before they started the task (i.e. at baseline) and after each block of the task. Our hypothesis was that the 2-back condition would induce more fatigue than the 0-back condition. However, this was not the case. While we found significant differences in fatigue between the groups (*F*(1,46) = 7.51, *p* = 0.009, *η^2^* = 0.14) and across the five fatigue ratings (*F*(4,414) = 3.77, *p* = 0.005, *η^2^* = 0.04), participants reported comparable amounts of fatigue on the two tasks (see figure 1). Initially, this surprised us; surely, we reasoned, the ‘harder’ task should induce more fatigue than the ‘easier’ task because the harder task should take more effort than the easier task. However, while the 2-back condition may be more difficult than the 0-back condition, it does not necessarily follow that it requires more effort to perform. This is because the 0-back condition is punishingly easy—that is, it is so easy that it may require considerable effort for participants to remain engaged. Thus, while the two tasks may differ in difficulty, they may nevertheless both require considerable effort to perform (though for different reasons), resulting in comparable fatigue.

**Figure 1.**
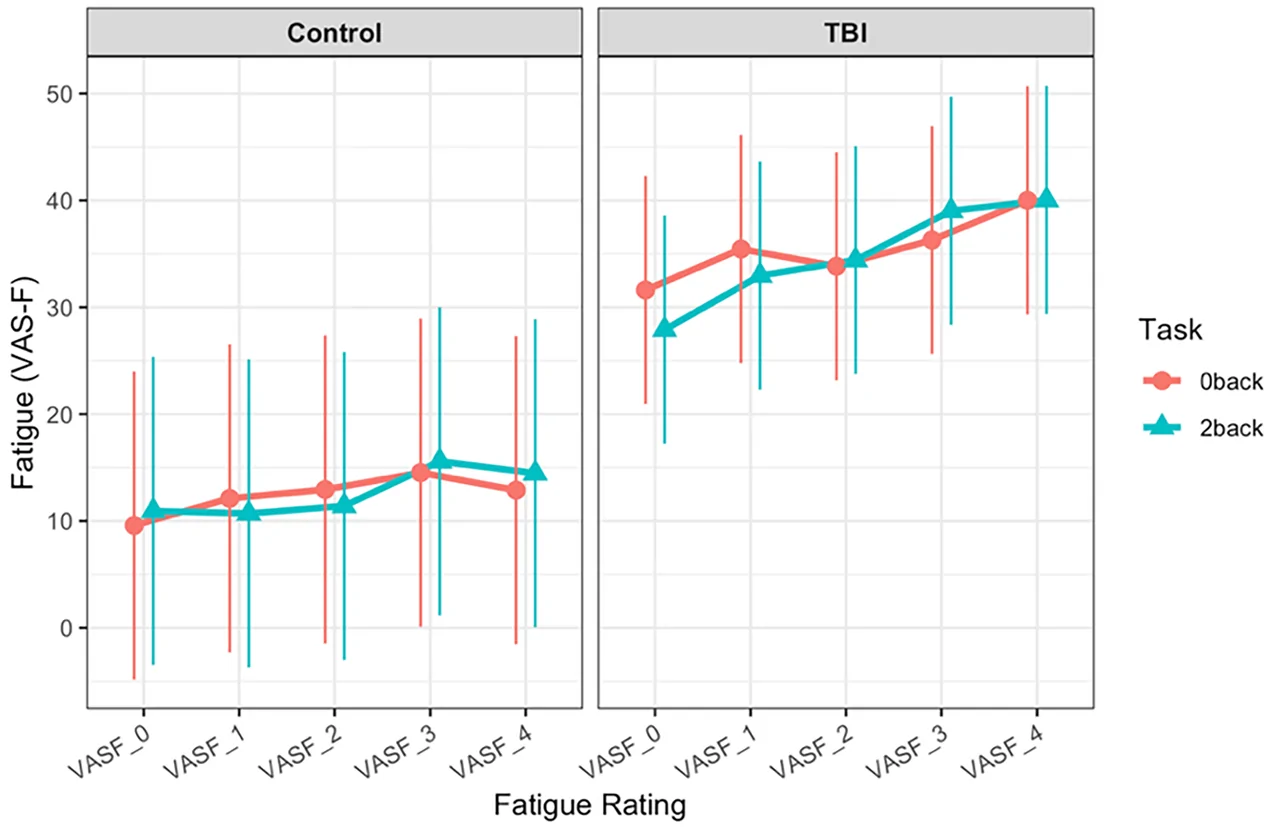
Fatigue ratings as a function of block. Participants' ratings on a VASF are shown across the blocks of the experiment for the 0-back task (red points) and for the 2-back task (blue triangles). For each task, the baseline rating is denoted as VASF_0 and the rating provided after each successive block is denoted by subsequent numbers (VASF_1, VASF_2, etc.). The error bars represent the 95% confidence interval.

There are several ways to assess the amount of effort required for a given task. Perhaps the simplest way is to ask participants to rate the amount of effort they expend to perform a given task. More sophisticated approaches have recently been developed in the field of neuroeconomics (e.g. [26]). Methods, such as effort discounting paradigms, allow one to assess the effort required for one condition of a task relative to another condition. This is often done by telling participants that they will have to perform a randomly determined condition of the task at a later time. Then, participants work through a series of trials in which they are asked to judge whether they would rather perform an easier condition of the task for one price (e.g. $2.00) or a more difficult version of the task for another, higher amount (e.g. $3.00) (e.g. [27,28]). By varying the amount offered for the more difficult version of the task, it is possible to determine the amount of money required to ‘make up for’ the difference in difficulty. This difference in monetary amount can be used as a quantitative, objective measure of the difference in effort required to perform the more difficult condition of the task. Operationalizing effort in this way holds promise as a useful tool to better understand fatigue.

The other side of the effort–reward balance is, of course, reward. Even effortful tasks can be undertaken with minimal fatigue if they are sufficiently rewarding, suggesting that reward is an important component of fatigue. As with interoception, there is a literature on reward. This literature has shown that the basal ganglia play a central role in the processing of rewards (e.g. [29,30]). This, when considered alongside the fatigue experienced by individuals with Parkinson's disease, offers converging evidence of the important role the basal ganglia play in fatigue processing. Indeed, we have shown that the fatigue experienced by individuals with multiple sclerosis (MS) [19,31–34] and TBI [25,34,35] is related to changes in activation in the caudate nucleus of the basal ganglia.

## 3. The balance between effort and reward

The evidence presented above suggests that fatigue is related to the balance between effort and reward. We and others have proposed that fatigue is a signal that the brain generates when the balance between effort and reward changes in such a way that the effort required no longer merits the reward received. This change can be dynamic, such as when fatigue develops as we work through a long series of tasks, or it can be static, such as when one has sustained brain injury or disease. Fatigue can develop dynamically when we repeatedly perform a given task because each time we do the task, we expend cognitive resources. If the reward we receive does not increase in a commensurate way, the balance between effort and reward gradually shifts such that the effort required to perform the task increases (because of decreasing cognitive resources) while the reward received does not. Fatigue may be the brain's way of signalling to itself that this shift away from homeostasis has occurred. Fatigue can also be static and is a persistent and debilitating symptom following brain injury (such as stroke or TBI) or disease (such as PD or MS). In these cases, neurological insult degrades neural signalling, thereby making virtually every aspect of cognition more effortful. For example, TBI causes damage to the long-range white matter fibres in the brain. These fibres support efficient neural signalling, and when they are damaged, the brain compensates by increasing short-range connections (e.g. [36]). In an analogous way, when a highway is closed, motorists compensate by using a network of local roads to reach their destinations. While this may be an effective strategy, it is not an efficient one; that is, it gets the job done, but the job takes more neural resources to complete. Or, to put it another way, the effort required is increased. If the reward received is not increased commensurately, then the effort–reward balance is persistently skewed such that the effort expended is rarely matched by the reward received, and (according to our model) persistent fatigue is the result.

This hypothesis is testable, and one way to test it relies on the psychophysical work done by Swets and collleagues in the 1950s (see [37,38]). Researchers found that by changing the effort–reward balance, they could change participants' response bias. In these experiments, participants were asked to detect a faint signal (e.g. a tone) in the presence of noise by pressing a button. In one condition, participants were told that they would receive a relatively large reward when they correctly detected the signal (e.g. $0.50) and that the penalty for making a false alarm (pressing the button when the signal was absent) was slight (e.g. a deduction of $0.10). In this case, participants adopted a liberal response bias, pressing the button whenever a signal might have been present, thereby maximizing their payments at the end of the experiment. In another condition, the costs and benefits of the ‘payoff matrix’ were reversed: correctly detecting the signal resulted in a modest reward (e.g. $0.10), whereas making a false alarm resulted in a large penalty (e.g. a deduction of $0.50). In this case, participants adopted a conservative response bias, responding only when they were sure that a signal had been presented. This pattern of results led to the development of a framework that allowed researchers to distinguish participants' response bias from their sensitivity to detecting the signal—a framework called Signal Detection Theory (SDT) [37].

To return to our hypothesis that fatigue is a signal the brain generates when the effort–reward balance changes in a way that homeostasis is jeopardized (and the effort no longer merits the reward), SDT provides a framework to test this idea because changes in the effort–reward balance should result in changes in response bias. We and others have tested this hypothesis both in the context of dynamic fatigue, showing that as participants become more fatigued, they also exhibit a more conservative response bias [39,40], and in the context of static fatigue, showing that individuals with MS report higher levels of fatigue than controls (even before undertaking a fatiguing task) and they also exhibit group differences in response bias—responding with a more conservative response bias [32]. These results lend support to our hypothesis about what fatigue is, which is the beginning of a mechanistic hypothesis of fatigue.

## 4. Studying fatigue

Fatigue has often been studied using ‘trait’ measures of fatigue. These are instruments that ask participants to report the extent to which they have been fatigued over a fairly long timespan in the recent past (e.g. over the past four weeks). These instruments are easy to administer and tend to correlate well with one another, suggesting that they assess similar constructs. However, it is less clear whether they are able to capture participants' fatigue over the timespan measured with fidelity, for at least two reasons. The first reason is that these instruments ask participants to rate other aspects of cognition. Examples include attention, arousal and memory. This may be understandable, inasmuch as fatigue can affect virtually every aspect of cognition; however, it results in a ‘fatigue score’ that is contaminated by ratings of aspects of cognition that may, for a given individual, be unrelated to fatigue. The second issue with trait measures of fatigue is that retrospective self-report measures of this kind are subject to several biases. For example, suppose we ask a participant to rate the following statement on a seven-point scale where 1 corresponds to ‘strongly disagree’ and 7 corresponds to ‘strongly agree’: ‘during the past week, I have been easily fatigued’. The participant responds with a 6. Does this mean that she has been easily fatigued each day during the past week? Does it mean that she was easily fatigued on most days? Does it mean that she has somehow computed an average of her instantaneous experience of fatigue over the past week, and that average corresponds to a rating of 6? It is very unclear. It is also unclear what other factors affect the rating (e.g. depression or poor sleep on the preceding night). For some applications, it may be enough to know that, for whatever reason, the participant felt that she had been easily fatigued in the past week. However, for a mechanistic understanding of fatigue, the uncertainty associated with this type of fatigue score is simply too great.

In our work (e.g. [25,41–47]) and in the work of others who seek a mechanistic understanding of fatigue (e.g. [48–52]), a different approach has been used. We induced fatigue using a task that requires a manageable level of effort and measured the fatigue that results. This approach has several benefits. Participants are asked to rate only their instantaneous experience of fatigue at the time the rating is made (‘state’ fatigue), thus reducing the extent to which extraneous factors affect their ratings. Indeed, they are explicitly told to avoid considering previous ratings. Additionally, participants' fatigue scores are directly tied to the fatigue-inducing task, facilitating the association of a particular fatigue rating with task performance on a particular block of trials. This has allowed us to show that, for example, increasing fatigue is associated with participants adopting an increasingly conservative response bias [32,40]. Furthermore, if physiological recordings are made during the fatigue induction task, it is possible to relate changes in physiology (e.g. brain activation or heart rate variability) to changes in fatigue (e.g. [53]). Using this logic, we have shown that brain activation in the insula, the basal ganglia and the anterior cingulate cortex (ACC) changes as a function of fatigue ratings. In fact, there appears to be a network of brain areas that are sensitive to fatigue: the insula, the basal ganglia, the ACC, the ventromedial prefrontal cortex, the dorsolateral prefrontal cortex and the thalamus (e.g. [14,17,34]).

While these areas have been shown to be sensitive to fatigue, the role each plays in the experience of fatigue is less clear. This is because most studies that have reported a relationship between activation in these areas and fatigue have relied on correlations. In an effort to better understand whether the basal ganglia play a causal role in fatigue, we recently induced fatigue in a group of stroke survivors who had sustained unilateral damage to their basal ganglia [54]. When compared to a group of matched controls or a matched group of stroke survivors who had sustained damage elsewhere in the brain, the group with basal ganglia damage showed a marked increase in the rate at which fatigue accrued. Because of the role that the basal ganglia play in monitoring effort and reward, we interpret this as converging evidence for the centrality of effort and reward in the experience of fatigue. In this case, when the ability to monitor effort and reward is compromised, fatigue may accrue unusually quickly because the brain conservatively ‘assumes’ a lack of homeostasis. That is, in the absence of robust signalling from the basal ganglia that the effort required merits the reward received, homeostasis is assumed to be at risk, and fatigue results.

Another line of converging evidence comes from an investigation into the role of the ACC in fatigue [46]. In this work, we tested the hypothesis that fatigue results from a violation of the expectation that the reward received would merit the effort required, and that this is similar to a violation of outcome prediction that participants experience when they make an error. The ACC responds robustly when participants respond erroneously, and this has been hypothesized to signal a mismatch between their expectation that they would respond correctly and their actual (erroneous) response (e.g. [55,56]). To test our hypothesis, we first replicated previous studies by showing a strong pattern of activation in the ACC associated with trials in which participants made an erroneous response. When we overlaid the pattern of activation associated with fatigue on the error-related activation pattern, we found clusters of voxels that responded to both fatigue and erroneous responding. This supported our hypothesis that fatigue may result from a violation of outcome prediction—in this case, the prediction of a balance between effort and reward.

## 5. Fatigue and functional neurological disorder

If our hypothesis about fatigue is correct, then fatigue is a domain-general mechanism designed to maintain homeostasis by encouraging a change in behaviour when the balance between effort and reward is disadvantageous. That is, when the effort expended no longer merits the reward received, homeostasis is lost and fatigue results. This conceptualization of fatigue suggests that there is no qualitative difference in the fatigue experienced by widely different populations. That is, the fatigue experienced following brain injury is expected to be qualitatively the same as the fatigue experienced after neurological disease, migraine, viral infection, or FND. If fatigue is a signal that the brain generates to tell itself that homeostasis has been compromised, then we might expect it to be the most common symptom in a disorder as heterogeneous as FND, and indeed, this is the case [1]. In fact, fatigue is more common in FND than any other single symptom, which is exactly what one would predict if fatigue is a domain-general signal produced when homeostasis is jeopardized.

Given the heterogeneity in the presentation of FND, it is perhaps unsurprising that investigations into the brain areas and/or brain networks associated with FND have produced mixed results [57,58]. In this respect, FND may be similar to TBI, which is caused by a wide range of events that produce idiosyncratic and highly variable damage to the brain. Despite this heterogeneity, fatigue is both prevalent and persistent following TBI [59,60], as it is in individuals with FND. In both cases, fatigue is likely owing to disruptions in homeostasis caused by inefficient processing rather than frank damage to brain areas that are sensitive to fatigue (see above). In the case of TBI, inefficient processing is owing to the structural damage resulting from TBI. In FND, the cause of inefficient processing is less clear, but deficits in information processing speed, attention and working memory are common [61], leading to more effortful processing [61] and, according to our model, a disruption of homeostasis.

The heterogeneity of FND is clearly challenging for researchers and clinicians; however, it also represents an opportunity, at least in relation to fatigue. If our model of fatigue is correct, then a reduction in effort should lead to a reduction in fatigue (if reward is held constant). Therefore, to the extent that inefficient, effortful processing can be characterized and isolated, it should be possible to alleviate fatigue by targeting the relevant processes. For example, if working memory is found to be effortful for a given patient, strategies designed to reduce working memory load should alleviate fatigue. Equally, the provision of rewards related to completing working memory tasks should also reduce fatigue. These rewards need not be tangible (e.g. monetary) in order to be reinforcing—as the gaming industry shows, earning points for achievements can be highly motivating. Alleviating fatigue by reducing the effort required or by increasing the reward received (or both) would have a strong positive effect on the quality of life for individuals with FND.

## 6. A way forward

The findings reviewed above suggest a way forward as we work to understand fatigue in FND. For example, rather than simply documenting that individuals with FND report high levels of trait fatigue, we should induce fatigue and document the resulting changes in state fatigue. This approach minimizes the self-report biases that plague trait fatigue measures. Furthermore, we should look for fatigue-related changes in brain activation in specific brain areas that have been shown to be sensitive to fatigue. These include the basal ganglia, the insula and the ACC, as well as the ventromedial and dorsolateral prefrontal cortices and the thalamus. Changes in activation in these areas allow us to understand more than simply that individuals with FND experience heightened levels of fatigue. The pattern of changes in brain activation will help us to understand how the mechanisms of fatigue are affected by FND. This is also true for behavioural metrics of fatigue, such as response bias. For example, given what we have shown in other populations [32], we would expect that the heightened levels of fatigue in FND would be related to a more conservative response bias.

As the tools available for studying fatigue expand and our understanding of fatigue becomes more sophisticated, my hope is that we will be able to develop a detailed, mechanistic model of fatigue in FND. I anticipate that this model would allow for the fractionation of fatigue into specific phenotypes—for example, individuals for whom the subjective experience of fatigue increases at a particularly fast rate on a particular type of task. Based on our findings in individuals who have survived a stroke that has damaged the basal ganglia [54], we might expect less basal ganglia activation in such individuals. Additionally, based on our finding that fatigue accrues at a faster rate in individuals whose ‘brain age’ is older than their chronological age (that is, the morphology of their brains more closely resembles people who are older than they are) [62], we would predict that an individual who fatigues more quickly than normal may have a less healthy, ‘older’-appearing brain. How other metrics of fatigue are affected in this putative phenotype is not yet known, and future research should include metrics of effort, manipulations of reward and investigations of changes in response bias. Additional metrics that currently show promise include heart rate variability, actigraphy and ecological momentary assessments. Metrics such as these, used in conjunction with state fatigue measures and neuroimaging, will allow for the development of a mechanistic model of fatigue in FND.

## 7. Conclusion

The tools of cognitive neuroscience, allied with more venerable tools, such as SDT, are helping us understand fatigue as never before. We are already well on the way to building a mechanistic model of fatigue in clinical populations, such as MS and TBI. The extent to which this model is able to explain fatigue in FND is not yet known, but it provides a promising starting point for understanding the fatigue that is so central to the symptomatology of FND. One lesson from the work that has been done to date is that fatigue seems to be a signal that the brain generates when the effort required no longer merits the reward received. There are many reasons for tasks to be effortful, and rewards come in many forms. Furthermore, different people find different things to be effortful and rewarding. Thus, fatigue is an umbrella term that has been used to describe what are likely to be several different phenotypes of fatigue. One of the exciting tasks before us is to delineate these different phenotypic manifestations of fatigue.

## Supplementary Material



## Data Availability

The data presented in figure 1 is provided in the supplementary material [63].

## Theme

One contribution of 12 to a discussion meeting issue ‘Digital healthcare for the management of functional neurological disorders’.
