# Supplementary material for: Towards a domain-general mechanistic model of fatigue: application to Functional Neurological Disorder
Source: Philos Trans R Soc Lond B Biol Sci. 2026 Sep 17;381(1958):2024-0462.1. doi: 10.1098/rstb.2024.0462 (PMC13644778; doi:10.1098/rstb.2024.0462)
Supplement: Supplementary file 1 [file rstb.2024.0462.zip › rstb.2024.0462/rstb20240462_si_001.docx]

**Supplementary Data**

Below is the data used for Figure 1 in the text. Subject_ID is an arbitrary code that was generated for each participant; Group indicates group membership (HC vs. TBI); Task indicates the task performed (0-back vs. 2-back); Time indicates when each fatigue rating was obtained (t0 indicates baseline, t1 indicates after block 1, t2 indicates after block2 and so on); VASF_score indicates the score provided at each timepoint, using a Visual Analogue Scale of Fatigue with a range from 0 to 100.

| Subject_ID | Group | Task | Time | VASF_score |
| --- | --- | --- | --- | --- |
| hc013 | hc | 2back | t0 | 5 |
| hc013 | hc | 2back | t1 | 5 |
| hc013 | hc | 2back | t2 | 5 |
| hc013 | hc | 2back | t3 | 20 |
| hc013 | hc | 2back | t4 | 30 |
| hc013 | hc | 0back | t0 | 20 |
| hc013 | hc | 0back | t1 | 40 |
| hc013 | hc | 0back | t2 | 50 |
| hc013 | hc | 0back | t3 | 50 |
| hc013 | hc | 0back | t4 | 50 |
| hc018 | hc | 2back | t0 | 0 |
| hc018 | hc | 2back | t1 | 10 |
| hc018 | hc | 2back | t2 | 12 |
| hc018 | hc | 2back | t3 | 10 |
| hc018 | hc | 2back | t4 | 5 |
| hc018 | hc | 0back | t0 | 0 |
| hc018 | hc | 0back | t1 | 0 |
| hc018 | hc | 0back | t2 | 0 |
| hc018 | hc | 0back | t3 | 0 |
| hc018 | hc | 0back | t4 | 0 |
| hc021 | hc | 2back | t0 | 0 |
| hc021 | hc | 2back | t1 | 0 |
| hc021 | hc | 2back | t2 | 0 |
| hc021 | hc | 2back | t3 | 0 |
| hc021 | hc | 2back | t4 | 1 |
| hc021 | hc | 0back | t0 | 1 |
| hc021 | hc | 0back | t1 | 1 |
| hc021 | hc | 0back | t2 | 2 |
| hc021 | hc | 0back | t3 | 2 |
| hc021 | hc | 0back | t4 | 2 |
| hc022 | hc | 2back | t0 | 5 |
| hc022 | hc | 2back | t1 | 5 |
| hc022 | hc | 2back | t2 | 5 |
| hc022 | hc | 2back | t3 | 5 |
| hc022 | hc | 2back | t4 | 5 |
| hc022 | hc | 0back | t0 | 5 |
| hc022 | hc | 0back | t1 | 5 |
| hc022 | hc | 0back | t2 | 5 |
| hc022 | hc | 0back | t3 | 5 |
| hc022 | hc | 0back | t4 | 5 |
| hc025 | hc | 2back | t0 | 10 |
| hc025 | hc | 2back | t1 | 10 |
| hc025 | hc | 2back | t2 | 10 |
| hc025 | hc | 2back | t3 | 12 |
| hc025 | hc | 2back | t4 | 12 |
| hc025 | hc | 0back | t0 | 12 |
| hc025 | hc | 0back | t1 | 12 |
| hc025 | hc | 0back | t2 | 15 |
| hc025 | hc | 0back | t3 | 15 |
| hc025 | hc | 0back | t4 | 15 |
| hc026 | hc | 2back | t0 | 0 |
| hc026 | hc | 2back | t1 | 0 |
| hc026 | hc | 2back | t2 | 0 |
| hc026 | hc | 2back | t3 | 10 |
| hc026 | hc | 2back | t4 | 10 |
| hc026 | hc | 0back | t0 | 0 |
| hc026 | hc | 0back | t1 | 10 |
| hc026 | hc | 0back | t2 | 15 |
| hc026 | hc | 0back | t3 | 20 |
| hc026 | hc | 0back | t4 | 20 |
| hc027 | hc | 2back | t0 | 10 |
| hc027 | hc | 2back | t1 | 5 |
| hc027 | hc | 2back | t2 | 10 |
| hc027 | hc | 2back | t3 | 40 |
| hc027 | hc | 2back | t4 | 40 |
| hc027 | hc | 0back | t0 | 40 |
| hc027 | hc | 0back | t1 | 30 |
| hc027 | hc | 0back | t2 | 10 |
| hc027 | hc | 0back | t3 | 10 |
| hc027 | hc | 0back | t4 | 5 |
| hc030 | hc | 2back | t0 | 10 |
| hc030 | hc | 2back | t1 | 20 |
| hc030 | hc | 2back | t2 | 20 |
| hc030 | hc | 2back | t3 | 20 |
| hc030 | hc | 2back | t4 | 20 |
| hc030 | hc | 0back | t0 | 20 |
| hc030 | hc | 0back | t1 | 20 |
| hc030 | hc | 0back | t2 | 20 |
| hc030 | hc | 0back | t3 | 30 |
| hc030 | hc | 0back | t4 | 20 |
| hc033 | hc | 2back | t0 | 0 |
| hc033 | hc | 2back | t1 | 20 |
| hc033 | hc | 2back | t2 | 15 |
| hc033 | hc | 2back | t3 | 20 |
| hc033 | hc | 2back | t4 | 15 |
| hc033 | hc | 0back | t0 | 10 |
| hc033 | hc | 0back | t1 | 15 |
| hc033 | hc | 0back | t2 | 25 |
| hc033 | hc | 0back | t3 | 15 |
| hc033 | hc | 0back | t4 | 15 |
| hc034 | hc | 2back | t0 | 60 |
| hc034 | hc | 2back | t1 | 0 |
| hc034 | hc | 2back | t2 | 0 |
| hc034 | hc | 2back | t3 | 0 |
| hc034 | hc | 2back | t4 | 0 |
| hc034 | hc | 0back | t0 | 0 |
| hc034 | hc | 0back | t1 | 0 |
| hc034 | hc | 0back | t2 | 0 |
| hc034 | hc | 0back | t3 | 0 |
| hc034 | hc | 0back | t4 | 0 |
| tbi002 | tbi | 2back | t0 | 0 |
| tbi002 | tbi | 2back | t1 | 0 |
| tbi002 | tbi | 2back | t2 | 5 |
| tbi002 | tbi | 2back | t3 | 10 |
| tbi002 | tbi | 2back | t4 | 10 |
| tbi002 | tbi | 0back | t0 | 0 |
| tbi002 | tbi | 0back | t1 | 40 |
| tbi002 | tbi | 0back | t2 | 20 |
| tbi002 | tbi | 0back | t3 | 5 |
| tbi002 | tbi | 0back | t4 | 5 |
| tbi003 | tbi | 2back | t0 | 10 |
| tbi003 | tbi | 2back | t1 | 10 |
| tbi003 | tbi | 2back | t2 | 10 |
| tbi003 | tbi | 2back | t3 | 20 |
| tbi003 | tbi | 2back | t4 | 10 |
| tbi003 | tbi | 0back | t0 | 10 |
| tbi003 | tbi | 0back | t1 | 10 |
| tbi003 | tbi | 0back | t2 | 50 |
| tbi003 | tbi | 0back | t3 | 20 |
| tbi003 | tbi | 0back | t4 | 60 |
| tbi005 | tbi | 2back | t0 | 0 |
| tbi005 | tbi | 2back | t1 | 0 |
| tbi005 | tbi | 2back | t2 | 0 |
| tbi005 | tbi | 2back | t3 | 0 |
| tbi005 | tbi | 2back | t4 | 0 |
| tbi005 | tbi | 0back | t0 | 0 |
| tbi005 | tbi | 0back | t1 | 0 |
| tbi005 | tbi | 0back | t2 | 0 |
| tbi005 | tbi | 0back | t3 | 0 |
| tbi005 | tbi | 0back | t4 | 0 |
| tbi006 | tbi | 2back | t0 | 20 |
| tbi006 | tbi | 2back | t1 | 40 |
| tbi006 | tbi | 2back | t2 | 50 |
| tbi006 | tbi | 2back | t3 | 50 |
| tbi006 | tbi | 2back | t4 | 60 |
| tbi006 | tbi | 0back | t0 | 60 |
| tbi006 | tbi | 0back | t1 | 70 |
| tbi006 | tbi | 0back | t2 | 70 |
| tbi006 | tbi | 0back | t3 | 80 |
| tbi006 | tbi | 0back | t4 | 80 |
| tbi010 | tbi | 2back | t0 | 10 |
| tbi010 | tbi | 2back | t1 | 30 |
| tbi010 | tbi | 2back | t2 | 30 |
| tbi010 | tbi | 2back | t3 | 40 |
| tbi010 | tbi | 2back | t4 | 50 |
| tbi010 | tbi | 0back | t0 | 50 |
| tbi010 | tbi | 0back | t1 | 40 |
| tbi010 | tbi | 0back | t2 | 50 |
| tbi010 | tbi | 0back | t3 | 50 |
| tbi010 | tbi | 0back | t4 | 50 |
| tbi013 | tbi | 2back | t0 | 50 |
| tbi013 | tbi | 2back | t1 | 85 |
| tbi013 | tbi | 2back | t2 | 95 |
| tbi013 | tbi | 2back | t3 | 98 |
| tbi013 | tbi | 2back | t4 | 99 |
| tbi013 | tbi | 0back | t0 | 95 |
| tbi013 | tbi | 0back | t1 | 99 |
| tbi013 | tbi | 0back | t2 | 99 |
| tbi013 | tbi | 0back | t3 | 99 |
| tbi013 | tbi | 0back | t4 | 100 |
| tbi014 | tbi | 2back | t0 | 0 |
| tbi014 | tbi | 2back | t1 | 0 |
| tbi014 | tbi | 2back | t2 | 0 |
| tbi014 | tbi | 2back | t3 | 70 |
| tbi014 | tbi | 2back | t4 | 70 |
| tbi014 | tbi | 0back | t0 | 70 |
| tbi014 | tbi | 0back | t1 | 50 |
| tbi014 | tbi | 0back | t2 | 0 |
| tbi014 | tbi | 0back | t3 | 0 |
| tbi014 | tbi | 0back | t4 | 0 |
| tbi018 | tbi | 2back | t0 | 50 |
| tbi018 | tbi | 2back | t1 | 50 |
| tbi018 | tbi | 2back | t2 | 50 |
| tbi018 | tbi | 2back | t3 | 50 |
| tbi018 | tbi | 2back | t4 | 50 |
| tbi018 | tbi | 0back | t0 | 50 |
| tbi018 | tbi | 0back | t1 | 50 |
| tbi018 | tbi | 0back | t2 | 50 |
| tbi018 | tbi | 0back | t3 | 50 |
| tbi018 | tbi | 0back | t4 | 50 |
| tbi022 | tbi | 2back | t0 | 20 |
| tbi022 | tbi | 2back | t1 | 60 |
| tbi022 | tbi | 2back | t2 | 60 |
| tbi022 | tbi | 2back | t3 | 80 |
| tbi022 | tbi | 2back | t4 | 90 |
| tbi022 | tbi | 0back | t0 | 50 |
| tbi022 | tbi | 0back | t1 | 100 |
| tbi022 | tbi | 0back | t2 | 80 |
| tbi022 | tbi | 0back | t3 | 90 |
| tbi022 | tbi | 0back | t4 | 100 |
| tbi023 | tbi | 2back | t0 | 10 |
| tbi023 | tbi | 2back | t1 | 0 |
| tbi023 | tbi | 2back | t2 | 5 |
| tbi023 | tbi | 2back | t3 | 5 |
| tbi023 | tbi | 2back | t4 | 5 |
| tbi023 | tbi | 0back | t0 | 10 |
| tbi023 | tbi | 0back | t1 | 10 |
| tbi023 | tbi | 0back | t2 | 5 |
| tbi023 | tbi | 0back | t3 | 20 |
| tbi023 | tbi | 0back | t4 | 20 |
| tbi025 | tbi | 2back | t0 | 10 |
| tbi025 | tbi | 2back | t1 | 0 |
| tbi025 | tbi | 2back | t2 | 20 |
| tbi025 | tbi | 2back | t3 | 20 |
| tbi025 | tbi | 2back | t4 | 0 |
| tbi025 | tbi | 0back | t0 | 0 |
| tbi025 | tbi | 0back | t1 | 0 |
| tbi025 | tbi | 0back | t2 | 0 |
| tbi025 | tbi | 0back | t3 | 0 |
| tbi025 | tbi | 0back | t4 | 0 |
| tbi026 | tbi | 2back | t0 | 60 |
| tbi026 | tbi | 2back | t1 | 40 |
| tbi026 | tbi | 2back | t2 | 20 |
| tbi026 | tbi | 2back | t3 | 0 |
| tbi026 | tbi | 2back | t4 | 0 |
| tbi026 | tbi | 0back | t0 | 20 |
| tbi026 | tbi | 0back | t1 | 0 |
| tbi026 | tbi | 0back | t2 | 0 |
| tbi026 | tbi | 0back | t3 | 0 |
| tbi026 | tbi | 0back | t4 | 0 |
| tbi029 | tbi | 2back | t0 | 10 |
| tbi029 | tbi | 2back | t1 | 20 |
| tbi029 | tbi | 2back | t2 | 20 |
| tbi029 | tbi | 2back | t3 | 20 |
| tbi029 | tbi | 2back | t4 | 30 |
| tbi029 | tbi | 0back | t0 | 30 |
| tbi029 | tbi | 0back | t1 | 30 |
| tbi029 | tbi | 0back | t2 | 40 |
| tbi029 | tbi | 0back | t3 | 70 |
| tbi029 | tbi | 0back | t4 | 75 |
| tbi030 | tbi | 2back | t0 | 70 |
| tbi030 | tbi | 2back | t1 | 60 |
| tbi030 | tbi | 2back | t2 | 80 |
| tbi030 | tbi | 2back | t3 | 90 |
| tbi030 | tbi | 2back | t4 | 100 |
| tbi030 | tbi | 0back | t0 | 100 |
| tbi030 | tbi | 0back | t1 | 100 |
| tbi030 | tbi | 0back | t2 | 100 |
| tbi030 | tbi | 0back | t3 | 100 |
| tbi030 | tbi | 0back | t4 | 100 |
| tbi032 | tbi | 2back | t0 | 70 |
| tbi032 | tbi | 2back | t1 | 70 |
| tbi032 | tbi | 2back | t2 | 90 |
| tbi032 | tbi | 2back | t3 | 100 |
| tbi032 | tbi | 2back | t4 | 100 |
| tbi032 | tbi | 0back | t0 | 90 |
| tbi032 | tbi | 0back | t1 | 100 |
| tbi032 | tbi | 0back | t2 | 100 |
| tbi032 | tbi | 0back | t3 | 100 |
| tbi032 | tbi | 0back | t4 | 100 |
| tbi033 | tbi | 2back | t0 | 50 |
| tbi033 | tbi | 2back | t1 | 60 |
| tbi033 | tbi | 2back | t2 | 60 |
| tbi033 | tbi | 2back | t3 | 70 |
| tbi033 | tbi | 2back | t4 | 90 |
| tbi033 | tbi | 0back | t0 | 60 |
| tbi033 | tbi | 0back | t1 | 90 |
| tbi033 | tbi | 0back | t2 | 90 |
| tbi033 | tbi | 0back | t3 | 90 |
| tbi033 | tbi | 0back | t4 | 90 |
| tbi034 | tbi | 2back | t0 | 50 |
| tbi034 | tbi | 2back | t1 | 80 |
| tbi034 | tbi | 2back | t2 | 0 |
| tbi034 | tbi | 2back | t3 | 10 |
| tbi034 | tbi | 2back | t4 | 0 |
| tbi034 | tbi | 0back | t0 | 30 |
| tbi034 | tbi | 0back | t1 | 30 |
| tbi034 | tbi | 0back | t2 | 20 |
| tbi034 | tbi | 0back | t3 | 10 |
| tbi034 | tbi | 0back | t4 | 15 |
| tbi035 | tbi | 2back | t0 | 0 |
| tbi035 | tbi | 2back | t1 | 0 |
| tbi035 | tbi | 2back | t2 | 0 |
| tbi035 | tbi | 2back | t3 | 0 |
| tbi035 | tbi | 2back | t4 | 10 |
| tbi035 | tbi | 0back | t0 | 0 |
| tbi035 | tbi | 0back | t1 | 10 |
| tbi035 | tbi | 0back | t2 | 10 |
| tbi035 | tbi | 0back | t3 | 5 |
| tbi035 | tbi | 0back | t4 | 5 |
| hc019 | hc | 0back | t0 | 0 |
| hc019 | hc | 0back | t1 | 0 |
| hc019 | hc | 0back | t2 | 0 |
| hc019 | hc | 0back | t3 | 0 |
| hc019 | hc | 0back | t4 | 0 |
| hc019 | hc | 2back | t0 | 0 |
| hc019 | hc | 2back | t1 | 0 |
| hc019 | hc | 2back | t2 | 0 |
| hc019 | hc | 2back | t3 | 0 |
| hc019 | hc | 2back | t4 | 0 |
| hc024 | hc | 0back | t0 | 2 |
| hc024 | hc | 0back | t1 | 0 |
| hc024 | hc | 0back | t2 | 0 |
| hc024 | hc | 0back | t3 | 1 |
| hc024 | hc | 0back | t4 | 1 |
| hc024 | hc | 2back | t0 | 2 |
| hc024 | hc | 2back | t1 | 2 |
| hc024 | hc | 2back | t2 | 2 |
| hc024 | hc | 2back | t3 | 2 |
| hc024 | hc | 2back | t4 | 2 |
| hc028 | hc | 0back | t0 | 0 |
| hc028 | hc | 0back | t1 | 0 |
| hc028 | hc | 0back | t2 | 0 |
| hc028 | hc | 0back | t3 | 0 |
| hc028 | hc | 0back | t4 | 2 |
| hc028 | hc | 2back | t0 | 0 |
| hc028 | hc | 2back | t1 | 1 |
| hc028 | hc | 2back | t2 | 1 |
| hc028 | hc | 2back | t3 | 2 |
| hc028 | hc | 2back | t4 | 2 |
| hc031 | hc | 0back | t0 | 3 |
| hc031 | hc | 0back | t1 | 3 |
| hc031 | hc | 0back | t2 | 3 |
| hc031 | hc | 0back | t3 | 4 |
| hc031 | hc | 0back | t4 | 4 |
| hc031 | hc | 2back | t0 | 4 |
| hc031 | hc | 2back | t1 | 4 |
| hc031 | hc | 2back | t2 | 4 |
| hc031 | hc | 2back | t3 | 4 |
| hc031 | hc | 2back | t4 | 4 |
| hc032 | hc | 0back | t0 | 10 |
| hc032 | hc | 0back | t1 | 10 |
| hc032 | hc | 0back | t2 | 10 |
| hc032 | hc | 0back | t3 | 15 |
| hc032 | hc | 0back | t4 | 15 |
| hc032 | hc | 2back | t0 | 15 |
| hc032 | hc | 2back | t1 | 25 |
| hc032 | hc | 2back | t2 | 25 |
| hc032 | hc | 2back | t3 | 30 |
| hc032 | hc | 2back | t4 | 30 |
| hc035 | hc | 0back | t0 | 10 |
| hc035 | hc | 0back | t1 | 20 |
| hc035 | hc | 0back | t2 | 30 |
| hc035 | hc | 0back | t3 | 40 |
| hc035 | hc | 0back | t4 | 30 |
| hc035 | hc | 2back | t0 | 30 |
| hc035 | hc | 2back | t1 | 40 |
| hc035 | hc | 2back | t2 | 50 |
| hc035 | hc | 2back | t3 | 50 |
| hc035 | hc | 2back | t4 | 50 |
| hc036 | hc | 0back | t0 | 30 |
| hc036 | hc | 0back | t1 | 40 |
| hc036 | hc | 0back | t2 | 35 |
| hc036 | hc | 0back | t3 | 40 |
| hc036 | hc | 0back | t4 | 35 |
| hc036 | hc | 2back | t0 | 35 |
| hc036 | hc | 2back | t1 | 35 |
| hc036 | hc | 2back | t2 | 35 |
| hc036 | hc | 2back | t3 | 40 |
| hc036 | hc | 2back | t4 | 20 |
| tbi001 | tbi | 0back | t0 | 0 |
| tbi001 | tbi | 0back | t1 | 0 |
| tbi001 | tbi | 0back | t2 | 0 |
| tbi001 | tbi | 0back | t3 | 0 |
| tbi001 | tbi | 0back | t4 | 0 |
| tbi001 | tbi | 2back | t0 | 0 |
| tbi001 | tbi | 2back | t1 | 0 |
| tbi001 | tbi | 2back | t2 | 0 |
| tbi001 | tbi | 2back | t3 | 0 |
| tbi001 | tbi | 2back | t4 | 0 |
| tbi004 | tbi | 0back | t0 | 0 |
| tbi004 | tbi | 0back | t1 | 0 |
| tbi004 | tbi | 0back | t2 | 0 |
| tbi004 | tbi | 0back | t3 | 0 |
| tbi004 | tbi | 0back | t4 | 0 |
| tbi004 | tbi | 2back | t0 | 0 |
| tbi004 | tbi | 2back | t1 | 0 |
| tbi004 | tbi | 2back | t2 | 0 |
| tbi004 | tbi | 2back | t3 | 0 |
| tbi004 | tbi | 2back | t4 | 0 |
| tbi007 | tbi | 0back | t0 | 100 |
| tbi007 | tbi | 0back | t1 | 60 |
| tbi007 | tbi | 0back | t2 | 60 |
| tbi007 | tbi | 0back | t3 | 60 |
| tbi007 | tbi | 0back | t4 | 60 |
| tbi007 | tbi | 2back | t0 | 60 |
| tbi007 | tbi | 2back | t1 | 60 |
| tbi007 | tbi | 2back | t2 | 70 |
| tbi007 | tbi | 2back | t3 | 70 |
| tbi007 | tbi | 2back | t4 | 60 |
| tbi008 | tbi | 0back | t0 | 0 |
| tbi008 | tbi | 0back | t1 | 0 |
| tbi008 | tbi | 0back | t2 | 0 |
| tbi008 | tbi | 0back | t3 | 5 |
| tbi008 | tbi | 0back | t4 | 0 |
| tbi008 | tbi | 2back | t0 | 0 |
| tbi008 | tbi | 2back | t1 | 0 |
| tbi008 | tbi | 2back | t2 | 0 |
| tbi008 | tbi | 2back | t3 | 0 |
| tbi008 | tbi | 2back | t4 | 0 |
| tbi011 | tbi | 0back | t0 | 50 |
| tbi011 | tbi | 0back | t1 | 65 |
| tbi011 | tbi | 0back | t2 | 80 |
| tbi011 | tbi | 0back | t3 | 90 |
| tbi011 | tbi | 0back | t4 | 90 |
| tbi011 | tbi | 2back | t0 | 90 |
| tbi011 | tbi | 2back | t1 | 90 |
| tbi011 | tbi | 2back | t2 | 95 |
| tbi011 | tbi | 2back | t3 | 95 |
| tbi011 | tbi | 2back | t4 | 95 |
| tbi012 | tbi | 0back | t0 | 30 |
| tbi012 | tbi | 0back | t1 | 50 |
| tbi012 | tbi | 0back | t2 | 20 |
| tbi012 | tbi | 0back | t3 | 40 |
| tbi012 | tbi | 0back | t4 | 60 |
| tbi012 | tbi | 2back | t0 | 50 |
| tbi012 | tbi | 2back | t1 | 70 |
| tbi012 | tbi | 2back | t2 | 70 |
| tbi012 | tbi | 2back | t3 | 70 |
| tbi012 | tbi | 2back | t4 | 70 |
| tbi015 | tbi | 0back | t0 | 0 |
| tbi015 | tbi | 0back | t1 | 0 |
| tbi015 | tbi | 0back | t2 | 0 |
| tbi015 | tbi | 0back | t3 | 0 |
| tbi015 | tbi | 0back | t4 | 0 |
| tbi015 | tbi | 2back | t0 | 0 |
| tbi015 | tbi | 2back | t1 | 1 |
| tbi015 | tbi | 2back | t2 | 1 |
| tbi015 | tbi | 2back | t3 | 1 |
| tbi015 | tbi | 2back | t4 | 1 |
| tbi016 | tbi | 0back | t0 | 0 |
| tbi016 | tbi | 0back | t1 | 0 |
| tbi016 | tbi | 0back | t2 | 0 |
| tbi016 | tbi | 0back | t3 | 0 |
| tbi016 | tbi | 0back | t4 | 0 |
| tbi016 | tbi | 2back | t0 | 0 |
| tbi016 | tbi | 2back | t1 | 1 |
| tbi016 | tbi | 2back | t2 | 1 |
| tbi016 | tbi | 2back | t3 | 1 |
| tbi016 | tbi | 2back | t4 | 1 |
| tbi019 | tbi | 0back | t0 | 0 |
| tbi019 | tbi | 0back | t1 | 0 |
| tbi019 | tbi | 0back | t2 | 0 |
| tbi019 | tbi | 0back | t3 | 10 |
| tbi019 | tbi | 0back | t4 | 0 |
| tbi019 | tbi | 2back | t0 | 0 |
| tbi019 | tbi | 2back | t1 | 0 |
| tbi019 | tbi | 2back | t2 | 25 |
| tbi019 | tbi | 2back | t3 | 20 |
| tbi019 | tbi | 2back | t4 | 30 |
| tbi024 | tbi | 0back | t0 | 0 |
| tbi024 | tbi | 0back | t1 | 30 |
| tbi024 | tbi | 0back | t2 | 20 |
| tbi024 | tbi | 0back | t3 | 40 |
| tbi024 | tbi | 0back | t4 | 50 |
| tbi024 | tbi | 2back | t0 | 40 |
| tbi024 | tbi | 2back | t1 | 50 |
| tbi024 | tbi | 2back | t2 | 60 |
| tbi024 | tbi | 2back | t3 | 70 |
| tbi024 | tbi | 2back | t4 | 80 |
| tbi027 | tbi | 0back | t0 | 75 |
| tbi027 | tbi | 0back | t1 | 60 |
| tbi027 | tbi | 0back | t2 | 75 |
| tbi027 | tbi | 0back | t3 | 75 |
| tbi027 | tbi | 0back | t4 | 80 |
| tbi027 | tbi | 2back | t0 | 85 |
| tbi027 | tbi | 2back | t1 | 85 |
| tbi027 | tbi | 2back | t2 | 80 |
| tbi027 | tbi | 2back | t3 | 90 |
| tbi027 | tbi | 2back | t4 | 90 |
| tbi031 | tbi | 0back | t0 | 0 |
| tbi031 | tbi | 0back | t1 | 5 |
| tbi031 | tbi | 0back | t2 | 10 |
| tbi031 | tbi | 0back | t3 | 16 |
| tbi031 | tbi | 0back | t4 | 50 |
| tbi031 | tbi | 2back | t0 | 50 |
| tbi031 | tbi | 2back | t1 | 60 |
| tbi031 | tbi | 2back | t2 | 70 |
| tbi031 | tbi | 2back | t3 | 60 |
| tbi031 | tbi | 2back | t4 | 40 |
| tbi036 | tbi | 0back | t0 | 0 |
| tbi036 | tbi | 0back | t1 | 0 |
| tbi036 | tbi | 0back | t2 | 0 |
| tbi036 | tbi | 0back | t3 | 0 |
| tbi036 | tbi | 0back | t4 | 0 |
| tbi036 | tbi | 2back | t0 | 0 |
| tbi036 | tbi | 2back | t1 | 0 |
| tbi036 | tbi | 2back | t2 | 0 |
| tbi036 | tbi | 2back | t3 | 0 |
| tbi036 | tbi | 2back | t4 | 0 |
